# Supplementary material for: Epitaxial growth of metal-organic framework nanosheets into single-crystalline orthogonal arrays
Source: Nat Commun. 2023 Sep 18;14:5780. doi: 10.1038/s41467-023-41517-x (PMC10507060; doi:10.1038/s41467-023-41517-x)
Supplement: Supplementary file 1 — Supplementary Information File [file 41467_2023_41517_MOESM1_ESM.pdf]

Supplementary information

Epitaxial growth of metal-organic framework nanosheets into single-crystalline orthogonal arrays

Yingying Zou[1], Chao Liu[1]*, Chaoqi Zhang[1], Ling Yuan[1], Jiaxin Li[1], Tong Bao[1], Guangfeng Wei[2]*, Jin Zou[3]*, Chengzhong Yu[1,4]*

[1] School of Chemistry and Molecular Engineering, East China Normal University; Shanghai 200241, P. R. China.

[2] Shanghai Key Laboratory of Chemical Assessment and Sustainability, School of Chemical Science and Engineering, Tongji University, Shanghai, 200092, P. R. China.

[3] Materials Engineering and Centre for Microscopy and Microanalysis, University of Queensland; Brisbane, Queensland 4072, Australia.

[4] Australian Institute for Bioengineering and Nanotechnology, The University of Queensland; Brisbane, QLD 4072, Australia.

*Corresponding author. Email: cliu@chem.ecnu.edu.cn (CL); weigf@tongji.edu.cn (GFW); j.zou@uq.edu.au (JZ); czyu@chem.ecnu.edu.cn; c.yu@uq.edu.au (CZY)

Supplementary Methods

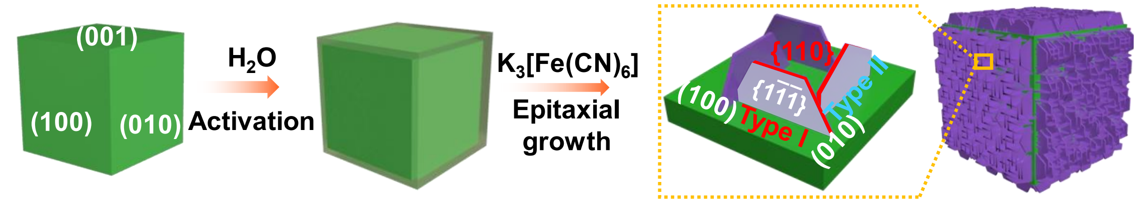

Supplementary Fig. 1 | Illustration of the formation process for the ONSA heterostructures.

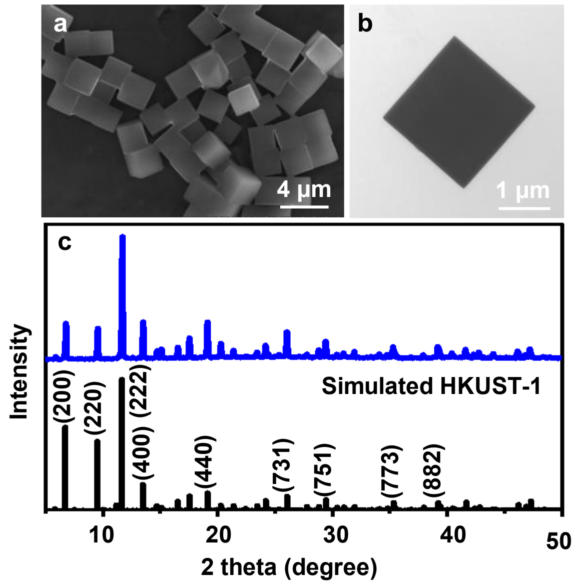

Supplementary Fig. 2 | Characterization of HKUST-1. a SEM and **b** TEM images, and **c** XRD pattern of HKUST-1.

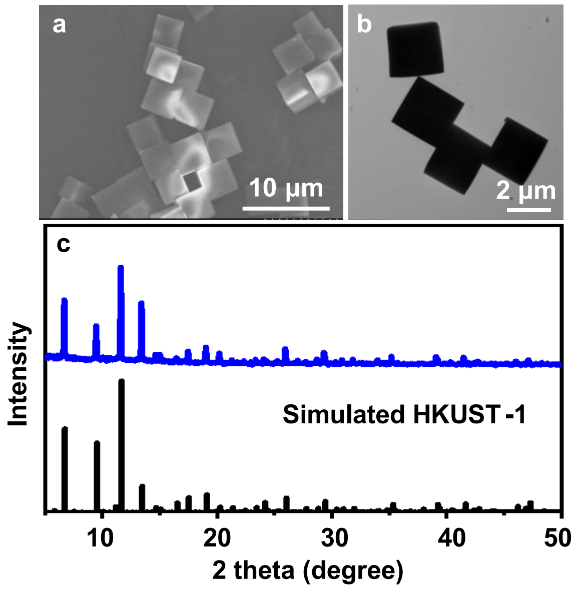

Supplementary Fig. 3 | Characterization of HKUST-1 after pre-activation. a SEM and

b TEM images, and **c** XRD pattern of HKUST-1 after pre-activation.

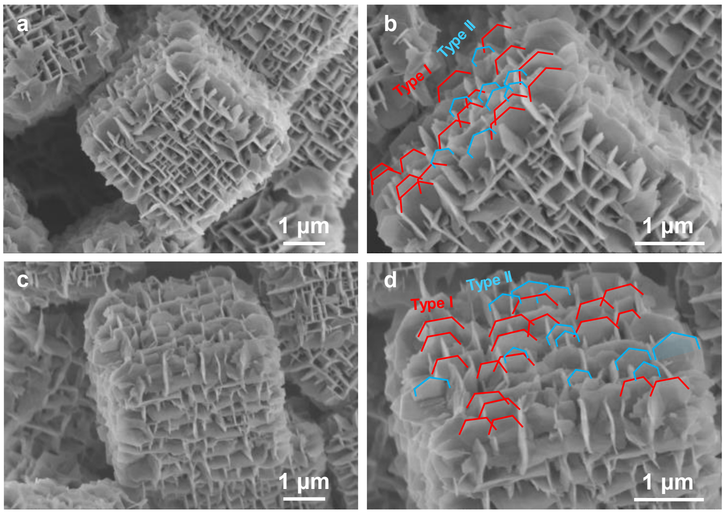

Supplementary Fig. 4 | The morphologies of nanosheets. The **a**, **c** SEM images and corresponding **b**, **d** enlarged SEM images of ONSA-HS-1.

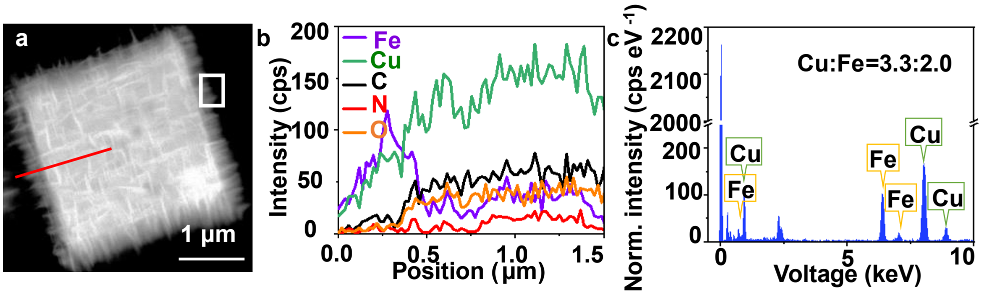

Supplementary Fig. 5 | The line and spot scanning spectrums of ONSA-HS-1. a STEM image of ONSA-HS-1, **b** line scanning spectra along the red line in **a**, **c** spot scan of white region in **a**. The red line and white rectangle in **a** represent the line scanning area and spot scanning area, respectively.

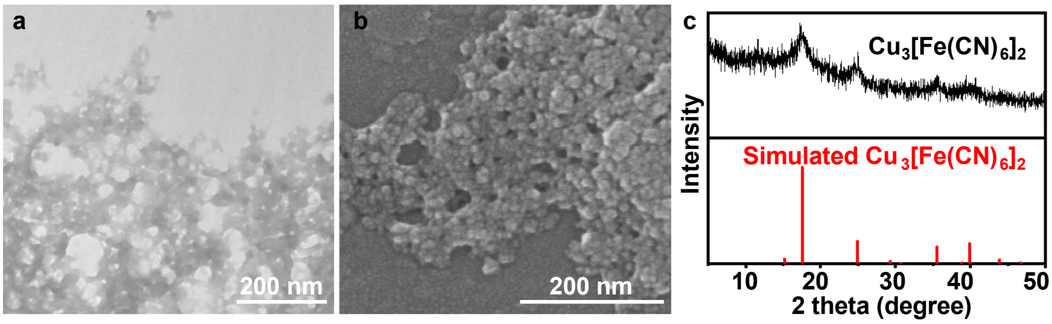

Supplementary Fig. 6 | Characterization of CuFe PBA nanocrystals. a TEM and **b** SEM images, and **c** XRD pattern of CuFe PBA by using $Cu(NO_3)_2 \cdot 3H_2O$ as the copper source.

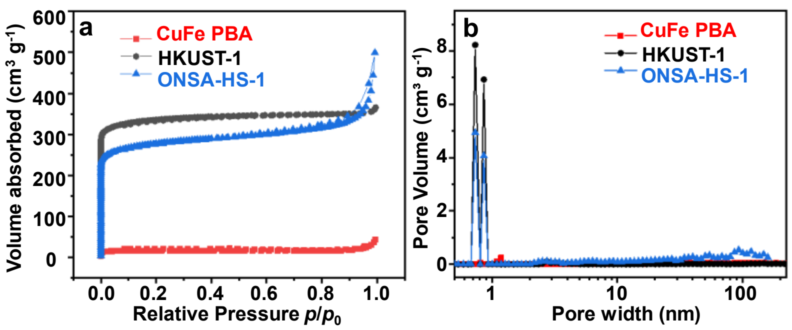

Supplementary Fig. 7 | Nitrogen adsorption-desorption isotherms. a Nitrogen adsorption-desorption isotherms (standard temperature and pressure) and **b** pore-size distribution curves (d*V*/dlog(*W*)) of HKUST-1, CuFe PBA and ONSA-HS-1.

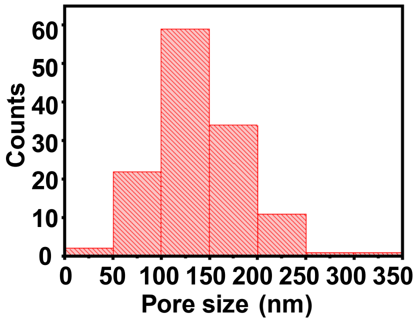

Supplementary Fig. 8 | Size distribution histogram of pores shown in Fig. 1**b**.

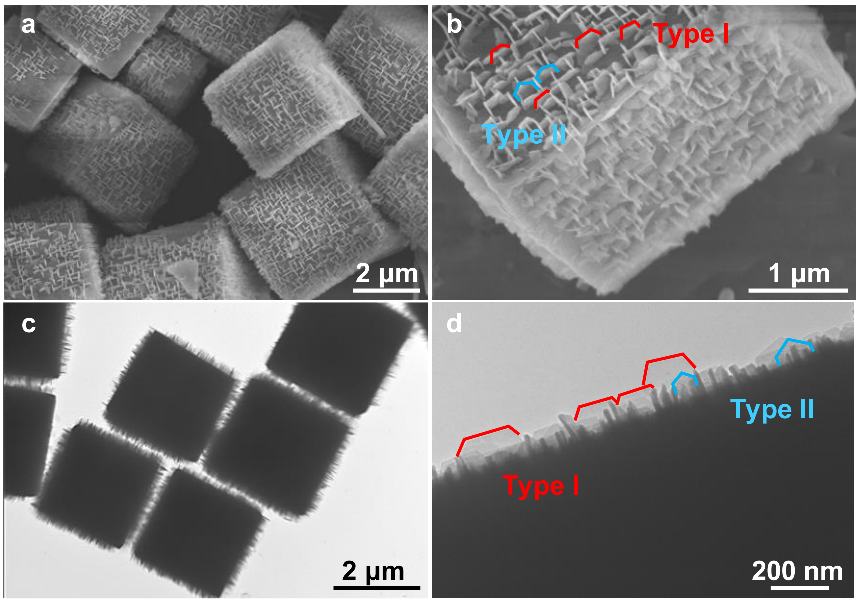

Supplementary Fig. 9 | The morphologies of ONSA-HS-1 at initial growth stage. a, **b** SEM and **c**, **d** TEM images of ONSA-HS-1 collected after growth of CuFe PBA for 10 min.

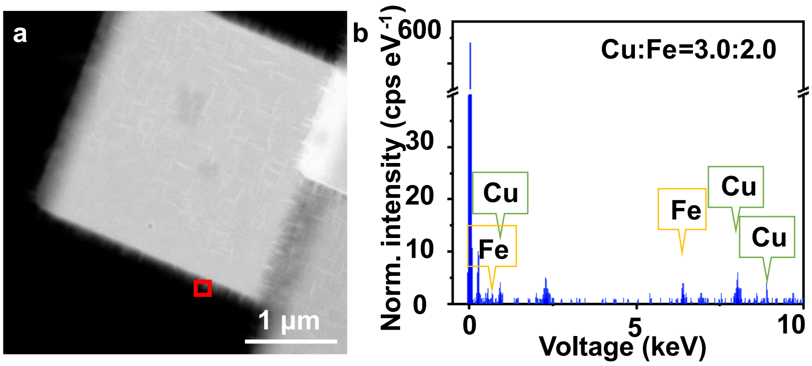

Supplementary Fig. 10 | The spot scanning spectra of ONSA-HS-1 at initial growth stage. a STEM image of ONSA-HS-1 collected after growth of CuFe PBA for 10 min and **b** spot scan of red region in **a**. The red rectangle in **a** represent the spot scanning area.

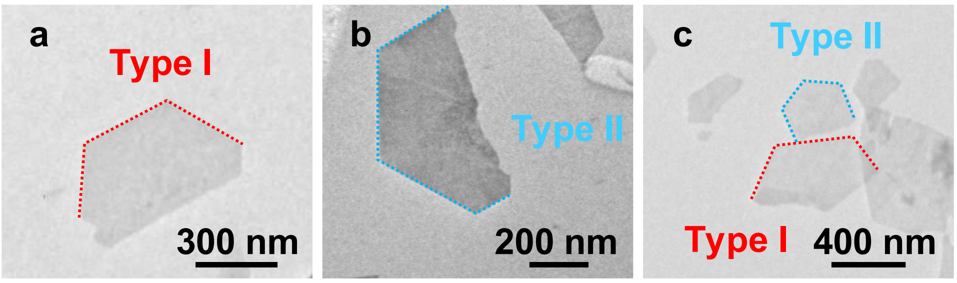

Supplementary Fig. 11 | The morphologies of detached CuFe PBA nanosheets. TEM images of detached CuFe PBA nanosheet with **a**, **c** Type I structure, **b**, **c** Type II structure.

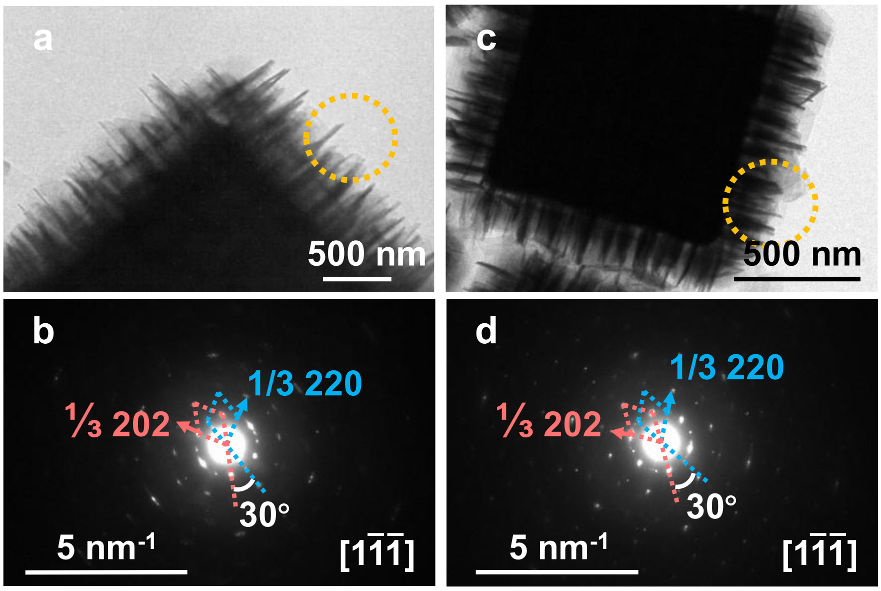

Supplementary Fig. 12 | The SAED patterns of ONSA-HS-1. a, **c** TEM images and corresponding **b**, **d** SAED patterns of the CuFe PBA nanosheets areas in ONSA-HS-1.

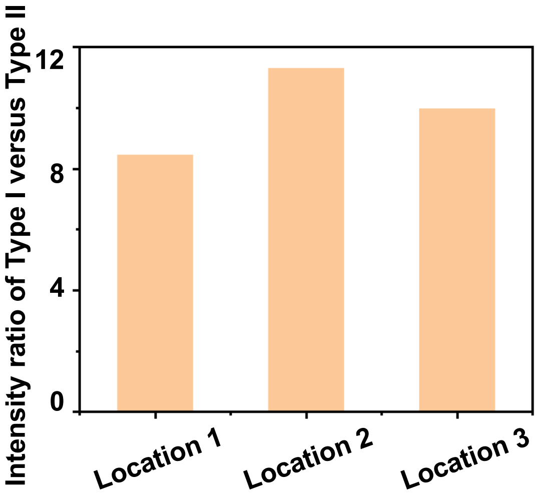

Supplementary Fig. 13 | The SAED spot intensity ratio of Type I versus Type II. The three locations were adapted from Fig. 2**f**, Supplementary Fig. 12**b** and Supplementary Fig. 12**d**, respectively.

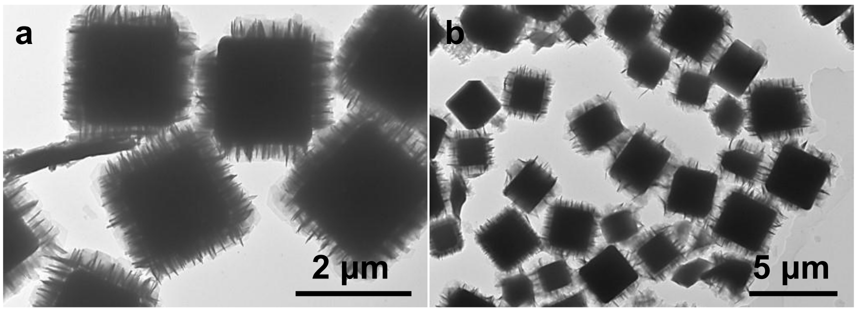

Supplementary Fig. 14 | The morphologies of ONSA-HS-2 and ONSA-HS-3. Low-magnification TEM images of **a** ONSA-HS-2 and **b** ONSA-HS-3.

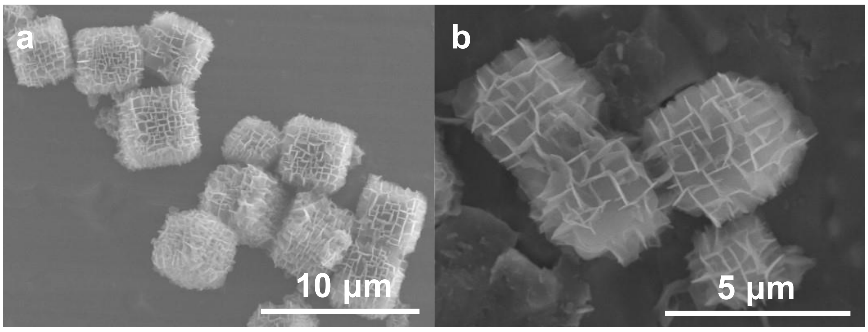

Supplementary Fig. 15 | The morphologies of ONSA-HS-2 and ONSA-HS-3. Low-magnification SEM images of **a** ONSA-HS-2 and **b** ONSA-HS-3.

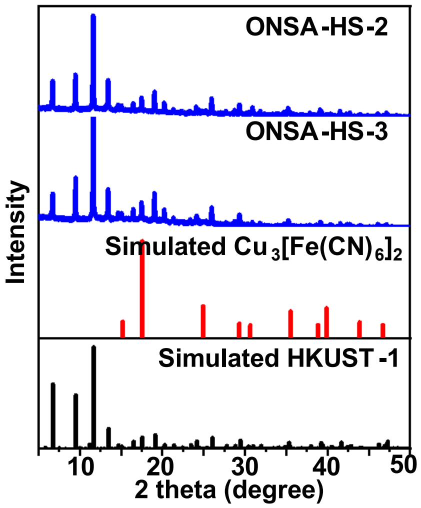

Supplementary Fig. 16 | XRD patterns of ONSA-HS-2 and ONSA-HS-3.

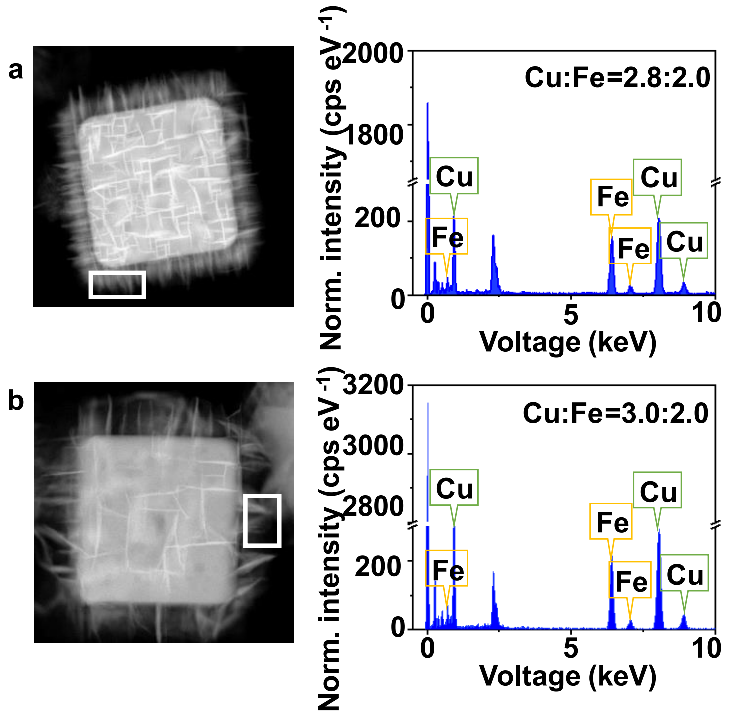

Supplementary Fig. 17 | The spot scanning spectrums of ONSA-HS-2 and ONSA-HS-3. STEM images and spot scanning spectra of white region of **a** ONSA-HS-2 and **b** ONSA-HS-3. The white rectangles in **a** and **b** represent the spot scanning areas of ONSA-HS-2 and ONSA-HS-3, respectively.

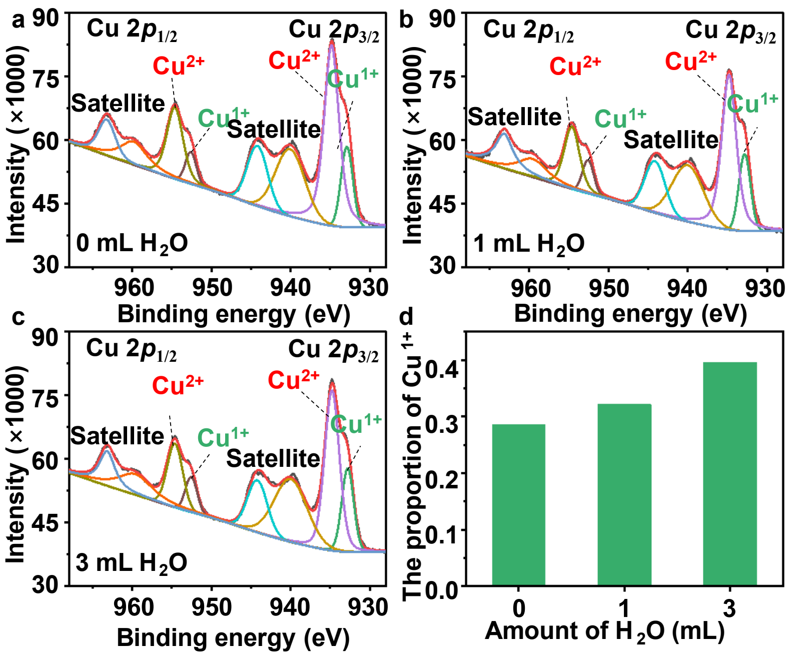

Supplementary Fig. 18 | XPS survey results of the HKUST-1 after activation. High-resolution XPS spectra of Cu 2*p* of HKUST-1 after activation by **a** 0, **b** 1 and **c** 3 mL of water, **d** the proportion of Cu^{+}/Cu^{2+}.

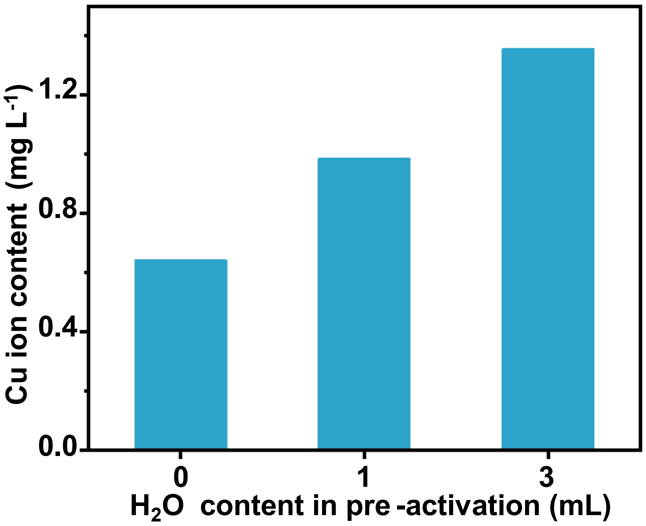

Supplementary Fig. 19 | Concentrations of copper ions in supernatants after activation of HKUST-1 by 0, 1 and 3 mL of H_2O.

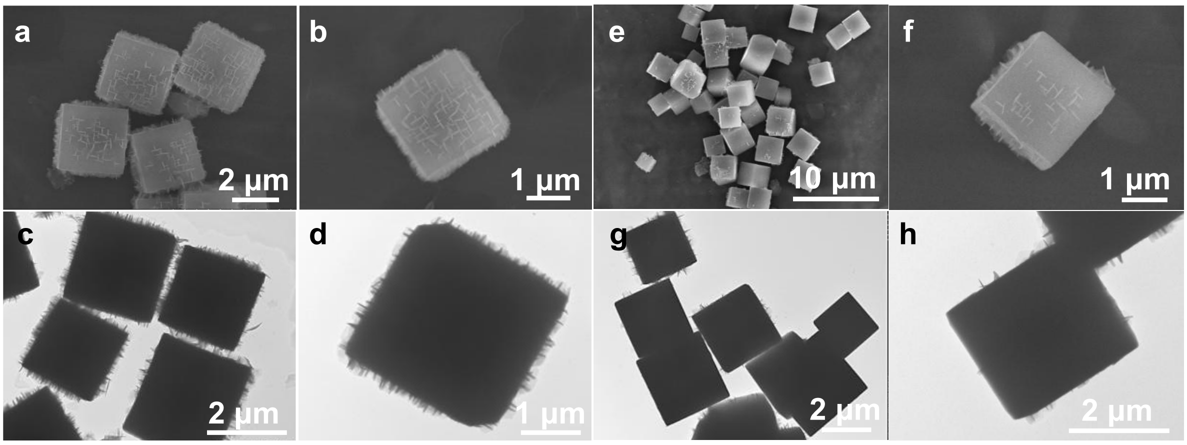

Supplementary Fig. 20 | The morphologies of ONSA-HS-2 and ONSA-HS-3. SEM and TEM images of **a-d** ONSA-HS-2 and **e-h** ONSA-HS-3 collected after growth of CuFe PBA for 10 min.

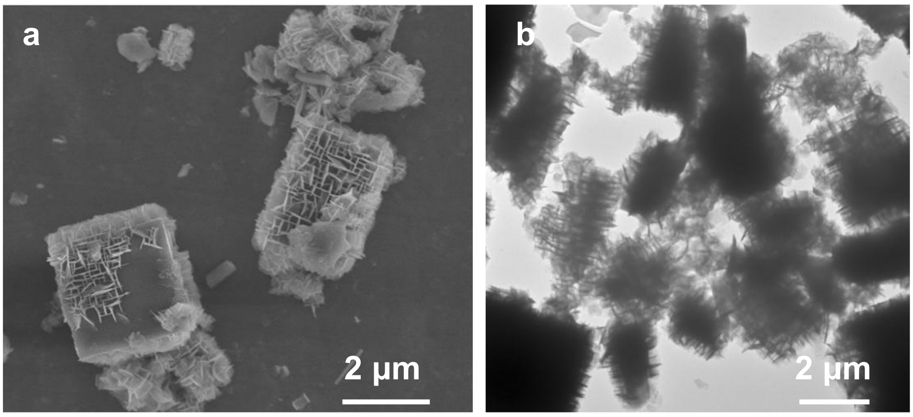

Supplementary Fig. 21 | The morphologies of ONSA-HS-4. a SEM and **b** TEM images of ONSA-HS-4.

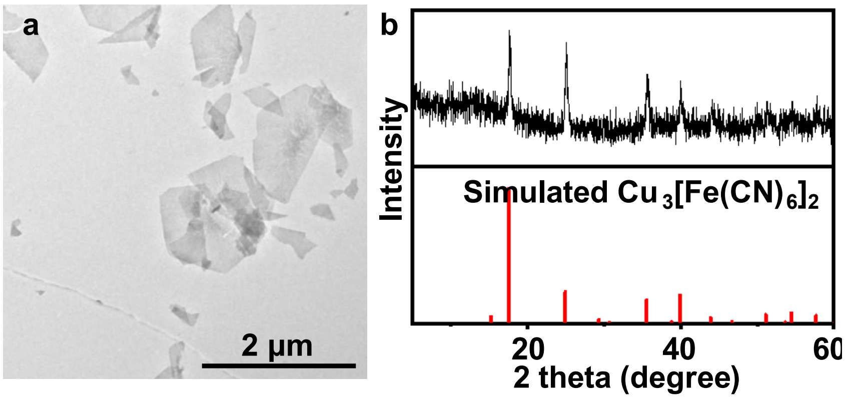

Supplementary Fig. 22 | Characterization of CuFe PBA nanosheets-111. a TEM images and **b** XRD pattern of collapsed CuFe PBA nanosheets (denoted as nanosheets-111).

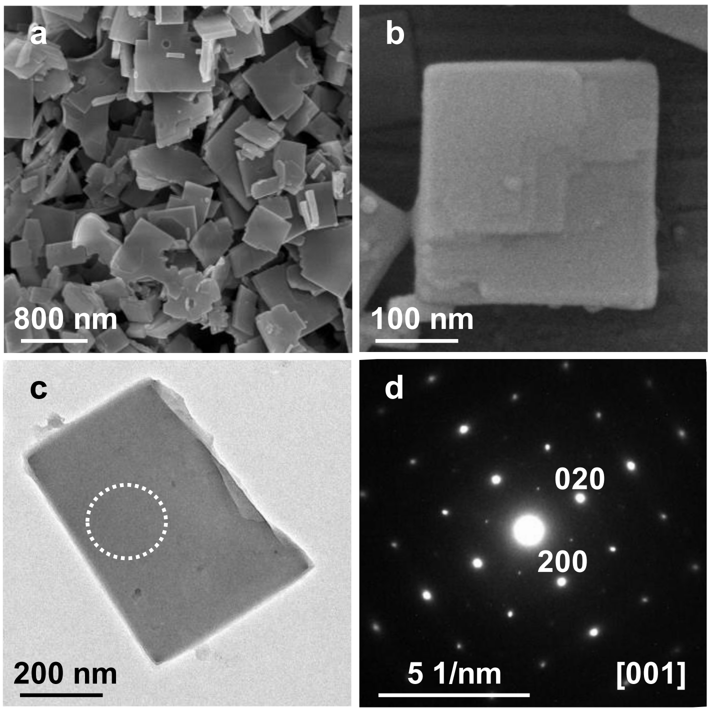

Supplementary Fig. 23 | Characterization of CuFe PBA nanosheets-001. a, **b** SEM images, **c** TEM image and corresponding **d** SAED pattern of CuFe PBA nanosheets-001.

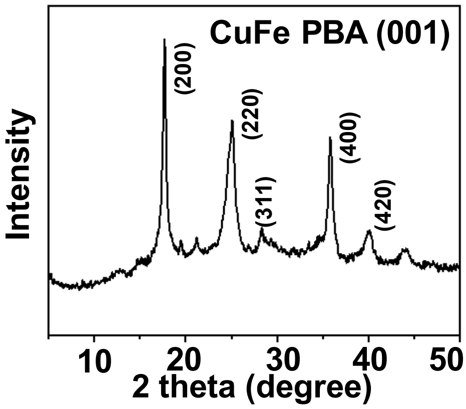

Supplementary Fig. 24 | XRD pattern of CuFe PBA nanosheets-001.

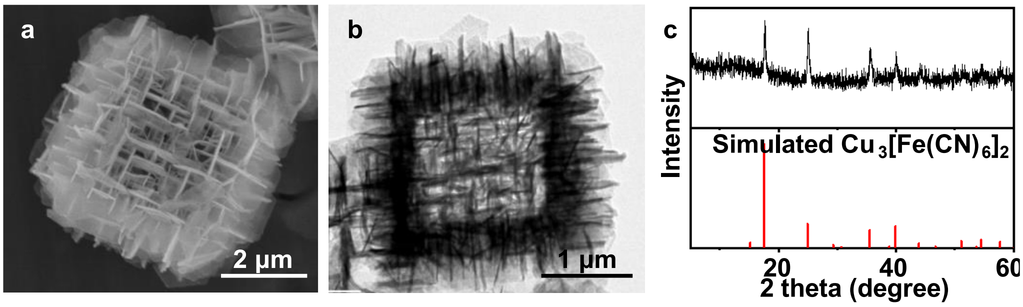

Supplementary Fig. 25 | Characterization of H-ONSA-A. a SEM and **b** TEM images, and **c** XRD pattern of H-ONSA-A.

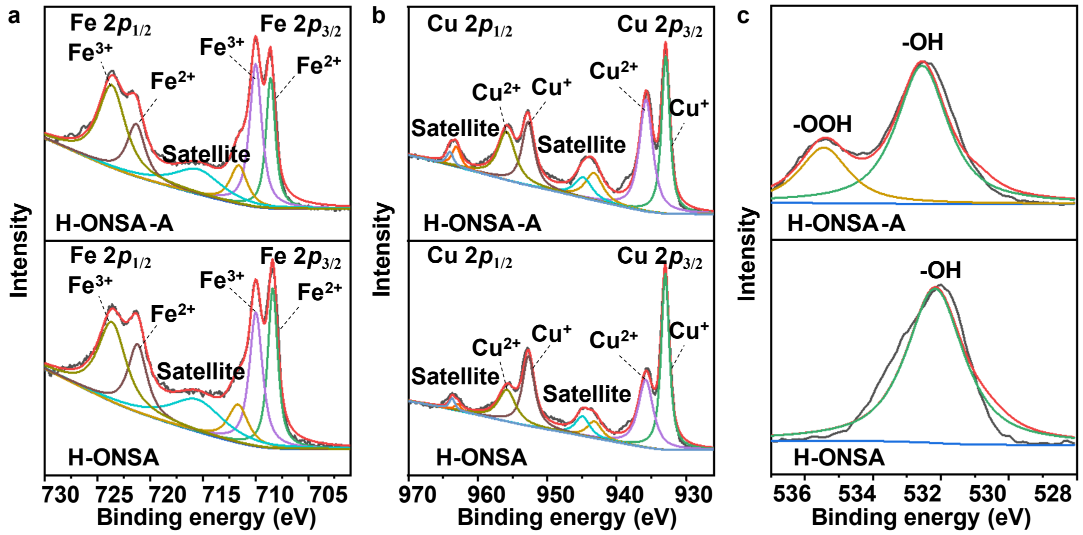

Supplementary Fig. 26 | XPS survey results of H-ONSA and H-ONSA-A. High-resolution XPS spectra of **a** Fe 2*p*, **b** Cu 2*p* and **c** O 1*s* of H-ONSA and H-ONSA-A.

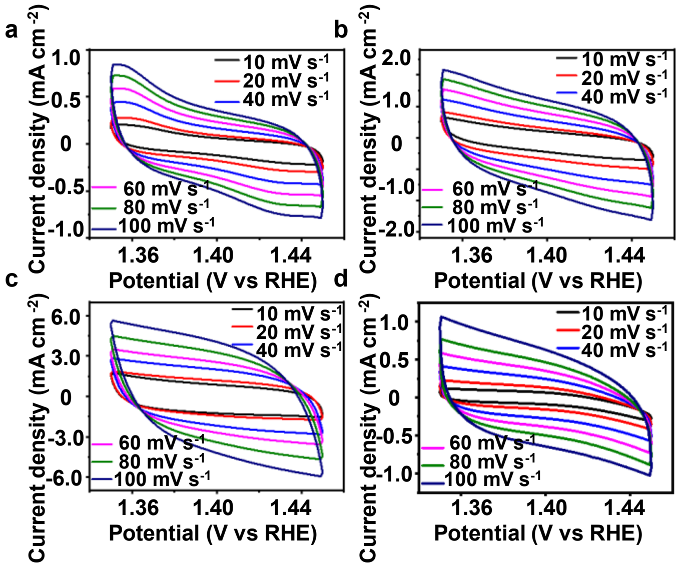

Supplementary Fig. 27 | CV curves of catalysts. CV curves between 1.35 and 1.45 V vs. RHE for **a** Nanocrystals, **b** Nanosheets-111, **c** H-ONSA and **d** Nanosheets-001.

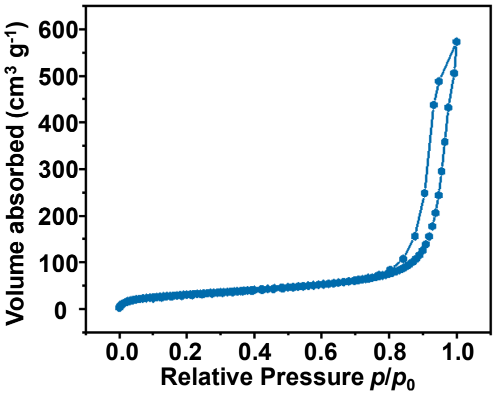

Supplementary Fig. 28 | Nitrogen adsorption-desorption isotherms (standard temperature

and pressure) of H-ONSA. The BET specific surface area is 113.4 m^2 g^{-1}.

Supplementary Table 1.

BET specific surface areas and pore volumes of HKUST-1, CuFe PBA and ONSA-HS-1.

Samples	BET specific surface area (m^2 g^{-1})	Pore volume (cm^3 g^{-1})
**HKUST-1**	1037	0.57
**CuFe PBA**	53	0.07
**ONSA-HS-1**	870	0.77

Supplementary Table 2.

Comparison of OER activity of H-ONSA and recently reported MOF-based catalysts in KOH solution.

Samples	Overpotential at 10 mA cm^{-2} (mV)	Tafel (mV dec^{-1})	Ref.
H-ONSA	241	52.1	**This work**
Co-MOF	320	142	1
NNU-23	365	81.8	2
Ni_2Fe_1 Sq-zbr-MOF	230	37.7	3
Ligand mixed MOF-Fe	288	39	4
NiFe‑MOFs	258	19	5
CoFe-PBA@NF-24	256	54	6
Ar-U-CoFe PBA	305	36.1	7
CoMoS	282	58	8
NiFe-DASC	310	45	9
meso-Fe-MoS_2/$CoMo_2S_4$	290	65	10
Ni-Co-Fe LDH nanosheet	288	92	11
$Co_{0.25}Fe_{0.75}O_4$	350	50	12
α-$Ni(OH)_2$	331	42	13
Ni-Fe mixed diselenide nanocages	240	24	14
NiCo@A-NiCo-PBA-AA	276	79.1	15
CoFe PBA/CoS_2-12 CNBs	265	59.2	16
O-GQD-NiFe PBA	259	52.5	17
NiCoFe-P-NP@ NiCoFe-PBA	223	78	18

PBA-5	271	53.7	19
H-SCP-350	291	38	20
CF-PBA-400	254	51	21
Co PBA-O/Ce-2	240	137	22
O-CNT/NiFe 1:18	279	42.8	23
NiFe-PBA	258	46	24
FeCoSx-PBA	266	33	25
P/Ni-Ag@AgCoPBA HHMCs	245	41.1	26

Supplementary references

1. Ma, T., Dai, S., Jaroniec, M., Qiao, S. Metal-organic framework derived hybrid Co_3O_4-carbon porous nanowire arrays as reversible oxygen evolution electrodes. *J. Am. Chem. Soc.* **136**, 13925 (2014).
2. Wang, X. et al. Exploring the performance improvement of the oxygen evolution reaction in a stable bimetal-organic framework system. *Angew. Chem. Int. Ed.* **57**, 9660-9664 (2018).
3. Kandambeth, S. et al. Unveiling chemically robust bimetallic squarate-based metal-organic frameworks for electrocatalytic oxygen evolution reaction. *Adv. Energy Mater.* **13**, 2202964 (2023).
4. Xue, Z. et al. Modulating electronic structure of metal-organic framework for efficient electrocatalytic oxygen evolution. *Adv. Energy Mater.* **8**, 1801564 (2018).
5. Wang, Y. et al. Engineering the activity and stability of MOF-nanocomposites for efficient water oxidation. *Adv. Energy Mater.* **11**, 2003759 (2021).
6. Chen, Z. et al. Ultrathin Prussian blue analogue nanosheet arrays with open bimetal centers for efficient overall water splitting. *Nano Energy* **68**, 104371 (2020).
7. Diao, F. et al. Moderate heat treatment of CoFe Prussian blue analogues for enhanced oxygen evolution reaction performance. *J. Energy Chem.* **78**, 476-486 (2023).
8. Guo, Y. et al. Multiscale structural optimization: Highly efficient hollow iron-doped metal sulfide heterostructures as bifunctional electrocatalysts for water splitting. *Nano Energy* **75**, 104913 (2020).
9. Zeng, Z. et al. Orbital coupling of hetero-diatomic nickel-iron site for bifunctional electrocatalysis of CO_2 reduction and oxygen evolution. *Nat. Commun.* **12**, 4088 (2021).
10. Guo, Y. et al. Mesoporous iron-doped MoS_2/$CoMo_2S_4$ heterostructures through organic-metal cooperative interactions on spherical micelles for electrochemical water splitting. *ACS Nano.* **14**, 4141-4152 (2020).
11. Zhang, M. Trimetallic NiCoFe-layered double hydroxides nanosheets efficient for oxygen evolution and highly selective oxidation of biomass-derived 5-hydroxymethylfurfural. *ACS Catal.* **10**, 5179 (2020).
12. Saddeler, S. et al. Influence of the cobalt content in cobalt iron oxides on the electrocatalytic OER activity. *J. Mater. Chem. A* **9**, 25381 (2021).
13. Gao, M. et al. Efficient water oxidation using nanostructured α-nickel-hydroxide as an electrocatalyst. *J. Am. Chem. Soc.* **136**, 7077-7084 (2014).
14. Nai, J., Lu, Y., Yu, L., Wang, X., Lou, X. W. Formation of Ni-Fe mixed diselenide nanocages as a superior oxygen evolution electrocatalyst. *Adv. Mater.* **29**, 1703870 (2017).
15. Zhang, H., Li, P., Chen, S., Xie, F., Riley, D. J. Anodic transformation of a core-shell prussian blue analogue to a bifunctional electrocatalyst for water splitting. *Adv. Funct. Mater.* 31, 2106835 (2021).
16. Xu, H. et al. Boosting electrocatalytic oxygen evolution over Prussian blue analog/transition metal dichalcogenide nanoboxes by photo-induced electron transfer. *J. Mater. Chem. A*, **7**, 26905-26910 (2019).
17. Lin, Y. et al. Graphene quantum dots induced defect-rich NiFe Prussian blue analogue as an efficient electrocatalyst for oxygen evolution reaction. *J. Colloid Interface Sci.*

648, 193-202 (2023).
18. Zhang, G. et al. In Situ Anchoring polymetallic phosphide nanoparticles within porous prussian blue analogue nanocages for boosting oxygen evolution catalysis. *Nano Lett.* **21**, 3016-3025 (2021).
19. Zhang, W. et al. Core-shell prussian blue analogs with compositional heterogeneity and open cages for oxygen evolution reaction. *Adv. Sci.* **6**, 1801901 (2019).
20. Wei, Y. et al. Preparation of hierarchical hollow CoFe Prussian blue analogues and its heat-treatment derivatives for the electrocatalyst of oxygen evolution reaction. *J. Colloid Interface Sci.* **631**, 8-16 (2023).
21. Zhou, J. et al. In situ exploring of the origin of the enhanced oxygen evolution reaction efficiency of metal(Co/Fe)-organic framework catalysts via postprocessing. *ACS Catal.* **12**, 3138-3148 (2022).
22. Kang L. et al. Dual-oxidation-induced lattice disordering in a Prussian blue analog for ultrastable oxygen evolution reaction performance. *J. Colloid Interface Sci.* **630**, 257-265 (2023).
23. Lin, Y., Chuang, C., Hsiao, L., Yeh, M., Ho, K. Oxygen plasma activation of carbon nanotubes-interconnected prussian blue analogue for oxygen evolution reaction. *ACS Appl. Mater. Interfaces*, **12**, 42634-42643 (2020).
24. Su, X. et al. Operando spectroscopic identification of active sites in NiFe prussian blue analogues as electrocatalysts: activation of oxygen atoms for oxygen evolution reaction. *J. Am. Chem. Soc.* **140**, 11286-11292 (2018).
25. Lu, M. et al. Electronic engineering of amorphous Fe-Co-S sites in hetero-nanoframes for oxygen evolution and flexible Al-air batteries. *J. Mater. Chem. A*, **10**, 19757-19768 (2022).
26. Zhang, H. et al. Construction of hierarchical P/Ni-Ag@AgCoPBA hollowstructures for boosting water oxidation activity. *Chem. Eng. J.* **446**, 137046 (2022).
